# Poly[[diaqua­bis­[μ-2-(4-fluoro­phen­oxy)acetato-κ^2^ *O* ^1^:*O* ^1′^]magnesium] 0.4-hydrate]

**DOI:** 10.1107/S1600536812035246

**Published:** 2012-08-15

**Authors:** Graham Smith

**Affiliations:** a Science and Engineering Faculty, Queensland University of Technology, GPO Box 2434, Brisbane, Queensland 4001, Australia

## Abstract

In the title compound, {[Mg(C_8_H_6_FO_3_)_2_(H_2_O)_2_]·0.4H_2_O}_*n*_, slightly distorted octa­hedral MgO_6_ complex units have crystallographic inversion symmetry, the coordination polyhedron comprising two *trans*-related water mol­ecules and four carboxyl O-atom donors, two of which are bridging. Within the two-dimensional complex polymer which is parallel to (100), coordinating water mol­ecules form inter­molecular O—H⋯O hydrogen bonds with carboxyl­ate and phen­oxy O-atom acceptors, as well as with the partial-occupancy solvent water mol­ecules.

## Related literature

For the structures of some magnesium complexes, derived from phen­oxy­acetic acids, see: Smith *et al.* (1980 ▶, 1981 ▶, 1982 ▶); Kennard *et al.* (1986 ▶). For the structures of other metal complexes with 4-fluoro­phen­oxy­acetate, see: O’Reilly *et al.* (1984 ▶); Smith *et al.* (1993 ▶).
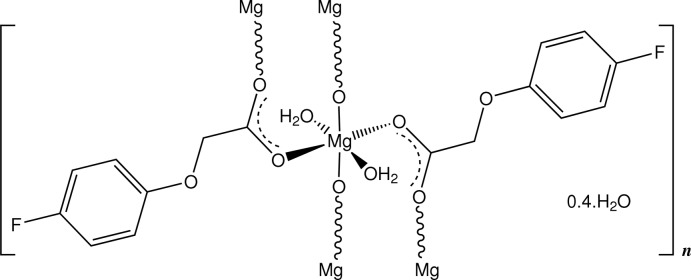


## Experimental

### 

#### Crystal data

[Mg(C_8_H_6_FO_3_)_2_(H_2_O)_2_]·0.4H_2_O
*M* *_r_* = 405.80
Monoclinic, 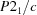

*a* = 17.2526 (9) Å
*b* = 6.8899 (3) Å
*c* = 7.5474 (3) Å
β = 95.118 (4)°
*V* = 893.57 (7) Å^3^
*Z* = 2
Mo *K*α radiation
μ = 0.17 mm^−1^
*T* = 200 K
0.30 × 0.20 × 0.05 mm

#### Data collection

Oxford Diffraction Gemini-S CCD-detector diffractometer
Absorption correction: multi-scan (*CrysAlis PRO*; Agilent, 2012 ▶) *T*_min_ = 0.964, *T*_max_ = 0.980
5825 measured reflections
1762 independent reflections
1400 reflections with *I* > 2σ(*I*)
*R*_int_ = 0.040

#### Refinement

*R*[*F* ^2^ > 2σ(*F* ^2^)] = 0.047
*wR*(*F* ^2^) = 0.109
*S* = 1.06
1762 reflections
133 parameters
H-atom parameters constrained
Δρ_max_ = 0.25 e Å^−3^
Δρ_min_ = −0.29 e Å^−3^

### 

Data collection: *CrysAlis PRO* (Agilent, 2012 ▶); cell refinement: *CrysAlis PRO*; data reduction: *CrysAlis PRO*; program(s) used to solve structure: *SIR92* (Altomare *et al.*, 1993 ▶); program(s) used to refine structure: *SHELXL97* (Sheldrick, 2008 ▶) within *WinGX* (Farrugia, 1999 ▶); molecular graphics: *PLATON* (Spek, 2009 ▶); software used to prepare material for publication: *PLATON*.

## Supplementary Material

Crystal structure: contains datablock(s) global, I. DOI: 10.1107/S1600536812035246/lh5512sup1.cif

Structure factors: contains datablock(s) I. DOI: 10.1107/S1600536812035246/lh5512Isup2.hkl

Supplementary material file. DOI: 10.1107/S1600536812035246/lh5512Isup3.cml

Additional supplementary materials:  crystallographic information; 3D view; checkCIF report

## Figures and Tables

**Table 1:** Selected bond lengths (Å)

Mg1—O1*W*	2.1032 (14)
Mg1—O21	2.0478 (14)
Mg1—O22^i^	2.0620 (14)

Symmetry code: (i) 
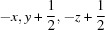
.

**Table 2:** Hydrogen-bond geometry (Å, °)

*D*—H⋯*A*	*D*—H	H⋯*A*	*D*⋯*A*	*D*—H⋯*A*
O1*W*—H11*W*⋯O1^iv^	0.91	2.45	3.214 (2)	143
O1*W*—H12*W*⋯O22^iv^	0.92	2.38	3.0352 (19)	128
O1*W*—H12*W*⋯O21^i^	0.92	1.92	2.760 (2)	151
O2*W*—H21*W*⋯O1^iv^	0.95	2.41	3.034 (10)	123
O2*W*—H22*W*⋯O22^iii^	0.85	2.13	2.950 (9)	160

Symmetry codes: (i) 
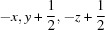
; (iii) 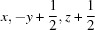; (iv) 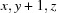.

**Fig. 1.**
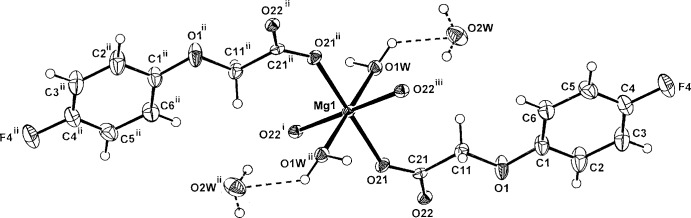
The molecular structure of the title compound, including the partial water molecules of solvation (O2W), with displacement ellipsoids drawn at the 50% probability level. For symmetry codes, see Table 1.

**Fig. 2.**
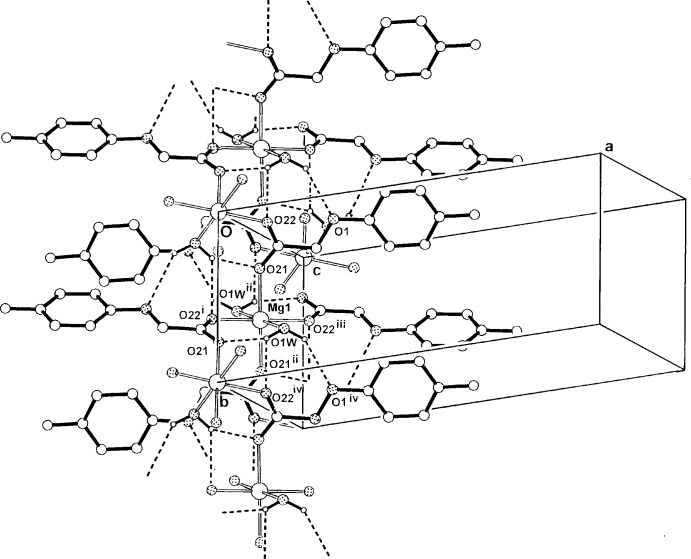
The hydrogen-bonding interactions, shown as dashed lines, in the title compound viewed along c. The partial water molecule of solvation and non-associative H-atoms have been omitted. For symmetry codes, see Tables 1 and 2.

## supplementary crystallographic information

### Comment

Magnesium complexes involving monoanionic phenoxyacetate ligands (*L*) show a variety of coordination modes, all based on an octahedral MgO_6_ metal stereochemistry, *e.g.* discrete monomeric [[Mg*L*_2_(H_2_O)_4_] (*L* = 2-fluorophenoxyacetate) (Kennard *et al.*, 1986); (*L* = 4-chloro-2-methylphenoxyacetate) (Smith *et al.*, 1981); [MgL(H_2_O)_5_] (*L* = 2,4,5-trichlorophenoxyacetate) (Smith *et al.*, 1982)], or polymeric [[Mg*L*_2_(H_2_O)_2_]_n_ (*L* = phenoxyacetate or 4-chlorophenoxyacetate) (Smith *et al.*, 1980)].

The title complex, [Mg(H_2_O)_2_(C_8_H_6_FO_3_)_2_]_n_ (0.4H_2_O)_n_ was obtained from the reaction of 4-fluorophenoxyacetic acid with MgCO_3_ in aqueous ethanol and the structure is reported herein. In this structure (Fig. 1), the slightly distorted octahedral MgO_6_ complex units [bond range Mg—O, 2.0478 (14)–2.1032 (14) Å (Table 1)] have crystallographic inversion symmetry, the coordination polyhedron comprising two *trans*-related water molecules and four carboxyl O-atom donors, two of which are bridging. Within the two-dimensional complex polymer layers which extend across (100), the coordinated water molecules from intermolecular O—H···O hydrogen-bonding interactions (Table 2), with carboxyl and phenoxy O-atom acceptors as well as with the partial water molecules of solvation (S.O.F. = 0.2) (Fig. 2). Except for the presence of the partial water molecules, the structure is similar to the those of the isomorphous Mg complexes with phenoxyacetate and 4-chlorophenoxyacetate (Smith *et al.*, 1980). In the present complex, the 4-fluorophenoxyacetate ligand is essentially planar, with the carboxyl group rotated slightly out of the plane [benzene ring to acetate dihedral angle = 12.26 (12)°].

### Experimental

The title compound was synthesized by the addition of excess MgCO_3_ to 15 ml of a hot aqueous ethanolic solution (10:1) of 4-fluorophenoxyacetic acid (0.1 g). After completion of the reaction, the excess MgCO_3_ was removed by filtration and the solution was allowed evaporate to incipient dryness at room temperature, giving thin colourless plates of the title compound from which a specimen was cleaved for the X-ray analysis.

### Refinement

Hydrogen atoms on the coordinated water molecule were located by difference methods and both positional and isotropic displacement parameters were initially refined but these were then allowed to ride, with *U*_iso_(H) = 1.5*U*_eq_(C). Other H-atoms were included in the refinement at calculated positions [C—H(aromatic) = 0.93 Å, 0.98 Å (methylene)] or O—H = 0.84–0.94 Å, with *U*_iso_(H) = 1.2*U*_eq_(C) or 1.5*U*_eq_(O), also using a riding-model approximation. The site occupancy factor for the partial water molecule of solvation was determined as 0.196 (4) and was subsequently fixed as 0.20.

### Crystal data

**Table:**

[Mg(C_8_H_6_FO_3_)_2_(H_2_O)_2_]·0.4H_2_O	*F*(000) = 420
*M**_r_* = 405.80	*D*_x_ = 1.508 Mg m^−^^3^
Monoclinic, *P*2_1_/*c*	Mo *K*α radiation, λ = 0.71073 Å
Hall symbol: -P 2ybc	Cell parameters from 1476 reflections
*a* = 17.2526 (9) Å	θ = 3.2–28.9°
*b* = 6.8899 (3) Å	µ = 0.17 mm^−^^1^
*c* = 7.5474 (3) Å	*T* = 200 K
β = 95.118 (4)°	Plate, colourless
*V* = 893.57 (7) Å^3^	0.30 × 0.20 × 0.05 mm
*Z* = 2	

### Data collection

**Table:**

Oxford Diffraction Gemini-S CCD-detector diffractometer	1762 independent reflections
Radiation source: Enhance (Mo) X-ray source	1400 reflections with *I* > 2σ(*I*)
Graphite monochromator	*R*_int_ = 0.040
Detector resolution: 16.077 pixels mm^-1^	θ_max_ = 26.0°, θ_min_ = 3.2°
ω scans	*h* = −21→21
Absorption correction: multi-scan (*CrysAlis PRO*; Agilent, 2012)	*k* = −8→8
*T*_min_ = 0.964, *T*_max_ = 0.980	*l* = −9→9
5825 measured reflections	

### Refinement

**Table:**

Refinement on *F*^2^	Primary atom site location: structure-invariant direct methods
Least-squares matrix: full	Secondary atom site location: difference Fourier map
*R*[*F*^2^ > 2σ(*F*^2^)] = 0.047	Hydrogen site location: inferred from neighbouring sites
*wR*(*F*^2^) = 0.109	H-atom parameters constrained
*S* = 1.06	*w* = 1/[σ^2^(*F*_o_^2^) + (0.0424*P*)^2^ + 0.393*P*] where *P* = (*F*_o_^2^ + 2*F*_c_^2^)/3
1762 reflections	(Δ/σ)_max_ < 0.001
133 parameters	Δρ_max_ = 0.25 e Å^−^^3^
0 restraints	Δρ_min_ = −0.29 e Å^−^^3^

### Special details

**Table:**

Geometry. Bond distances, angles *etc*. have been calculated using the rounded fractional coordinates. All su's are estimated from the variances of the (full) variance-covariance matrix. The cell e.s.d.'s are taken into account in the estimation of distances, angles and torsion angles
Refinement. Refinement of *F*^2^ against ALL reflections. The weighted *R*-factor *wR* and goodness of fit *S* are based on *F*^2^, conventional *R*-factors *R* are based on *F*, with *F* set to zero for negative *F*^2^. The threshold expression of *F*^2^ > σ(*F*^2^) is used only for calculating *R*-factors(gt) *etc*. and is not relevant to the choice of reflections for refinement. *R*-factors based on *F*^2^ are statistically about twice as large as those based on *F*, and *R*- factors based on ALL data will be even larger.

### Fractional atomic coordinates and isotropic or equivalent isotropic displacement parameters (Å^2^)

**Table:**

	*x*	*y*	*z*	*U*_iso_*/*U*_eq_	Occ. (<1)
Mg1	0.00000	0.50000	0.50000	0.0187 (3)	
F4	0.49577 (8)	−0.0237 (3)	0.7819 (2)	0.0567 (6)	
O1	0.20100 (10)	−0.0103 (3)	0.4489 (2)	0.0481 (6)	
O1W	0.09125 (8)	0.6177 (2)	0.36712 (19)	0.0255 (5)	
O21	0.01967 (8)	0.2303 (2)	0.39916 (19)	0.0255 (4)	
O22	0.07774 (8)	0.02846 (19)	0.22234 (18)	0.0220 (4)	
C1	0.27377 (13)	−0.0045 (4)	0.5408 (3)	0.0358 (8)	
C2	0.32768 (15)	−0.1323 (4)	0.4814 (4)	0.0519 (10)	
C3	0.40282 (15)	−0.1370 (4)	0.5616 (4)	0.0487 (9)	
C4	0.42218 (13)	−0.0158 (4)	0.7001 (3)	0.0393 (8)	
C5	0.37063 (15)	0.1118 (4)	0.7620 (3)	0.0408 (9)	
C6	0.29510 (14)	0.1183 (4)	0.6810 (3)	0.0378 (8)	
C11	0.14596 (12)	0.1286 (3)	0.4960 (3)	0.0264 (7)	
C21	0.07588 (12)	0.1271 (3)	0.3609 (3)	0.0194 (6)	
O2W	0.2262 (5)	0.5768 (14)	0.5816 (12)	0.048 (3)	0.200
H2	0.31330	−0.21540	0.38700	0.0620*	
H3	0.43940	−0.22160	0.52140	0.0580*	
H5	0.38580	0.19330	0.85700	0.0490*	
H6	0.25920	0.20490	0.72100	0.0450*	
H11A	0.16940	0.25660	0.50070	0.0320*	
H11B	0.12990	0.09860	0.61290	0.0320*	
H11W	0.13290	0.67830	0.42470	0.0380*	
H12W	0.06600	0.68980	0.27780	0.0380*	
H21W	0.22790	0.70540	0.62850	0.0710*	0.200
H22W	0.18990	0.52540	0.63450	0.0710*	0.200

### Atomic displacement parameters (Å^2^)

**Table:**

	*U*^11^	*U*^22^	*U*^33^	*U*^12^	*U*^13^	*U*^23^
Mg1	0.0232 (5)	0.0158 (5)	0.0167 (5)	0.0015 (4)	0.0003 (4)	−0.0012 (4)
F4	0.0271 (7)	0.0734 (12)	0.0671 (11)	0.0020 (8)	−0.0101 (7)	0.0008 (9)
O1	0.0388 (10)	0.0607 (12)	0.0412 (11)	0.0274 (9)	−0.0169 (8)	−0.0299 (9)
O1W	0.0261 (8)	0.0252 (8)	0.0249 (8)	0.0015 (7)	0.0008 (6)	0.0034 (6)
O21	0.0299 (8)	0.0197 (7)	0.0261 (8)	0.0051 (7)	−0.0011 (6)	−0.0065 (6)
O22	0.0274 (8)	0.0198 (7)	0.0186 (7)	0.0013 (6)	0.0003 (6)	−0.0028 (6)
C1	0.0310 (12)	0.0464 (15)	0.0286 (13)	0.0124 (12)	−0.0048 (10)	−0.0082 (11)
C2	0.0461 (16)	0.0655 (19)	0.0416 (16)	0.0250 (15)	−0.0107 (13)	−0.0223 (14)
C3	0.0373 (15)	0.0621 (18)	0.0457 (16)	0.0206 (14)	−0.0021 (12)	−0.0062 (14)
C4	0.0249 (12)	0.0519 (16)	0.0401 (15)	−0.0001 (12)	−0.0021 (11)	0.0062 (13)
C5	0.0354 (14)	0.0475 (16)	0.0383 (15)	−0.0048 (12)	−0.0037 (11)	−0.0096 (12)
C6	0.0334 (13)	0.0466 (15)	0.0327 (13)	0.0060 (12)	−0.0012 (11)	−0.0109 (12)
C11	0.0293 (12)	0.0258 (11)	0.0237 (11)	0.0069 (10)	−0.0005 (9)	−0.0071 (9)
C21	0.0254 (11)	0.0130 (9)	0.0198 (10)	−0.0022 (9)	0.0022 (8)	0.0013 (8)
O2W	0.045 (5)	0.054 (6)	0.043 (5)	−0.017 (5)	0.001 (4)	0.009 (4)

### Geometric parameters (Å, º)

**Table:**

Mg1—O1W	2.1032 (14)	O2W—H21W	0.9500
Mg1—O21	2.0478 (14)	C1—C2	1.384 (4)
Mg1—O22^i^	2.0620 (14)	C1—C6	1.379 (3)
Mg1—O1W^ii^	2.1032 (14)	C2—C3	1.381 (4)
Mg1—O21^ii^	2.0478 (14)	C3—C4	1.356 (4)
Mg1—O22^iii^	2.0620 (14)	C4—C5	1.363 (4)
F4—C4	1.362 (3)	C5—C6	1.390 (3)
O1—C1	1.380 (3)	C11—C21	1.511 (3)
O1—C11	1.416 (3)	C2—H2	0.9300
O21—C21	1.257 (3)	C3—H3	0.9300
O22—C21	1.250 (3)	C5—H5	0.9300
O1W—H11W	0.9100	C6—H6	0.9300
O1W—H12W	0.9200	C11—H11B	0.9700
O2W—H22W	0.8500	C11—H11A	0.9700
			
O1W—Mg1—O21	90.96 (5)	C1—C2—C3	120.3 (3)
O1W—Mg1—O22^i^	92.03 (5)	C2—C3—C4	118.7 (2)
O1W—Mg1—O1W^ii^	180.00	F4—C4—C3	118.7 (2)
O1W—Mg1—O21^ii^	89.04 (5)	F4—C4—C5	118.7 (2)
O1W—Mg1—O22^iii^	87.97 (5)	C3—C4—C5	122.6 (2)
O21—Mg1—O22^i^	84.33 (5)	C4—C5—C6	119.0 (2)
O1W^ii^—Mg1—O21	89.04 (5)	C1—C6—C5	119.6 (2)
O21—Mg1—O21^ii^	180.00	O1—C11—C21	109.90 (17)
O21—Mg1—O22^iii^	95.67 (5)	O21—C21—C11	115.38 (19)
O1W^ii^—Mg1—O22^i^	87.97 (5)	O22—C21—C11	119.32 (18)
O21^ii^—Mg1—O22^i^	95.67 (5)	O21—C21—O22	125.3 (2)
O22^i^—Mg1—O22^iii^	180.00	C3—C2—H2	120.00
O1W^ii^—Mg1—O21^ii^	90.96 (5)	C1—C2—H2	120.00
O1W^ii^—Mg1—O22^iii^	92.03 (5)	C2—C3—H3	121.00
O21^ii^—Mg1—O22^iii^	84.33 (5)	C4—C3—H3	121.00
C1—O1—C11	117.06 (19)	C4—C5—H5	120.00
Mg1—O21—C21	139.08 (14)	C6—C5—H5	121.00
Mg1^iv^—O22—C21	132.00 (13)	C5—C6—H6	120.00
Mg1—O1W—H12W	103.00	C1—C6—H6	120.00
H11W—O1W—H12W	114.00	O1—C11—H11A	110.00
Mg1—O1W—H11W	123.00	O1—C11—H11B	110.00
H21W—O2W—H22W	102.00	C21—C11—H11B	110.00
O1—C1—C6	124.9 (2)	H11A—C11—H11B	108.00
C2—C1—C6	119.9 (2)	C21—C11—H11A	110.00
O1—C1—C2	115.2 (2)		
			
O1W—Mg1—O21—C21	36.0 (2)	C6—C1—C2—C3	−0.2 (4)
O22^i^—Mg1—O21—C21	127.9 (2)	O1—C1—C6—C5	−179.4 (2)
O1W^ii^—Mg1—O21—C21	−144.0 (2)	C2—C1—C6—C5	−0.3 (4)
O22^iii^—Mg1—O21—C21	−52.1 (2)	C1—C2—C3—C4	0.7 (4)
C11—O1—C1—C2	−175.1 (2)	C2—C3—C4—F4	178.5 (2)
C11—O1—C1—C6	4.0 (3)	C2—C3—C4—C5	−0.7 (4)
C1—O1—C11—C21	169.15 (19)	F4—C4—C5—C6	−179.0 (2)
Mg1—O21—C21—O22	−136.10 (18)	C3—C4—C5—C6	0.2 (4)
Mg1—O21—C21—C11	43.1 (3)	C4—C5—C6—C1	0.3 (4)
Mg1^iv^—O22—C21—O21	4.0 (3)	O1—C11—C21—O21	172.84 (18)
Mg1^iv^—O22—C21—C11	−175.24 (13)	O1—C11—C21—O22	−7.9 (3)
O1—C1—C2—C3	179.0 (2)		

Symmetry codes: (i) −*x*, *y*+1/2, −*z*+1/2; (ii) −*x*, −*y*+1, −*z*+1; (iii) *x*, −*y*+1/2, *z*+1/2; (iv) −*x*, *y*−1/2, −*z*+1/2.

### Hydrogen-bond geometry (Å, º)

**Table:**

*D*—H···*A*	*D*—H	H···*A*	*D*···*A*	*D*—H···*A*
O1*W*—H11*W*···O1^v^	0.91	2.45	3.214 (2)	143
O1*W*—H12*W*···O22^v^	0.92	2.38	3.0352 (19)	128
O1*W*—H12*W*···O21^i^	0.92	1.92	2.760 (2)	151
O2*W*—H21*W*···O1^v^	0.95	2.41	3.034 (10)	123
O2*W*—H22*W*···O22^iii^	0.85	2.13	2.950 (9)	160

Symmetry codes: (i) −*x*, *y*+1/2, −*z*+1/2; (iii) *x*, −*y*+1/2, *z*+1/2; (v) *x*, *y*+1, *z*.

## References

[bb1] Agilent (2012). *CrysAlis PRO* Agilent Technologies Ltd, Yarnton, England.

[bb2] Altomare, A., Cascarano, G., Giacovazzo, C. & Guagliardi, A. (1993). *J. Appl. Cryst.* **26**, 343–350.

[bb3] Farrugia, L. J. (1999). *J. Appl. Cryst.* **32**, 837–838.

[bb4] Kennard, C. H. L., O’Reilly, E. J., Schiller, S., Smith, G. & White, A. H. (1986). *Aust. J. Chem.* **39**, 1823–1832.

[bb5] O’Reilly, E. J., Smith, G. & Kennard, C. H. L. (1984). *Inorg. Chim. Acta*, **90**, 63–71.

[bb6] Sheldrick, G. M. (2008). *Acta Cryst.* A**64**, 112–122. 10.1107/S0108767307043930 18156677

[bb7] Smith, G., Lynch, D. E., Mak, T. C. W., Yip, W.-H. & Kennard, C. H. L. (1993). *Polyhedron*, **12**, 203–208.

[bb8] Smith, G., O’Reilly, E. J. & Kennard, C. H. L. (1980). *J. Chem. Soc. Dalton Trans.* pp. 2462–2466.

[bb9] Smith, G., O’Reilly, E. J. & Kennard, C. H. L. (1981). *Cryst. Struct. Commun.* **10**, 1397–1402.

[bb10] Smith, G., O’Reilly, E. J. & Kennard, C. H. L. (1982). *Inorg. Chim. Acta*, **62**, 241–246.

[bb11] Spek, A. L. (2009). *Acta Cryst.* D**65**, 148–155. 10.1107/S090744490804362X PMC2631630 19171970

